# Inhibitors of Intracellular Signaling Pathways that Lead to Stimulated Epidermal Pigmentation: Perspective of Anti-Pigmenting Agents

**DOI:** 10.3390/ijms15058293

**Published:** 2014-05-12

**Authors:** Genji Imokawa, Koichi Ishida

**Affiliations:** 1 Research Institute for Biological Functions, Chubu University, 1200 Matsumoto, Kasugai, Aichi 487-8501, Japan; 2 Global R&D-Skin Beauty, Kao Corporation, Tokyo 103-8210, Japan; E-Mail: ishida.koichi@kao.co.jp

**Keywords:** intracellular signaling pathway, signaling inhibitors, endothelin, stem cell factor, ultraviolet B**-**melanosis, solar lentigo, melasma

## Abstract

Few anti-pigmenting agents have been designed and developed according to their known hyperpigmentation mechanisms and corresponding intracellular signaling cascades. Most anti-pigmenting agents developed so far are mechanistically involved in the interruption of constitutional melanogenic mechanisms by which skin color is maintained at a normal and unstimulated level. Thus, owing to the difficulty of confining topical application to a specific hyperpigmented skin area, potent anti-pigmenting agents capable of attenuating the natural unstimulated pigmentation process have the risk of leading to hypopigmentation. Since intracellular signaling pathways within melanocytes do not function substantially in maintaining normal skin color and are activated only by environmental stimuli such as UV radiation, specifically down-regulating the activation of melanogenesis to the constitutive level would be an appropriate strategy to develop new potent anti-pigmenting agents with a low risk of hypopigmentation. In this article, we review the hyperpigmentation mechanisms and intracellular signaling pathways that lead to the stimulation of melanogenesis. We also discuss a screening and evaluation system to select candidates for new anti-melanogenic substances by focusing on inhibitors of endothelin-1 or stem cell factor-triggered intracellular signaling cascades. From this viewpoint, we show that extracts of the herbs *Withania somnifera* and *Melia toosendan* and the natural chemicals *Withaferin* A and *Astaxanthin* are new candidates for potent anti-pigmenting substances that avoid the risk of hypopigmentation.

## 1. Introduction

Hyperpigmentary disorders in the medical cosmetic field include ultraviolet B (UVB) hyperpigmentation, melasma and solar lentigo, whose biological mechanisms, including the intrinsic melanogenic paracrine cytokines involved, have been well elucidated. Also, the corresponding intracellular signaling cascades that lead to the stimulation of melanogenesis in human melanocytes have been well implicated. However, few anti-pigmenting agents have been designed and developed according to their known hyperpigmentation mechanisms and corresponding intracellular signaling cascades. Most anti-melanogenic agents developed so far inhibit tyrosinase activity [1], stimulate the proteolytic degradation of tyrosinase through the proteasome system [2] and/or disrupt the transfer of melanin granules from melanocytes to keratinocytes via inactivation of the proteinase-activated receptor 2 (PAR2) on the plasma membrane of keratinocytes [3]. Those mechanisms have serious potential risks as follows: Even in normal skin color (non-hyperpigmented) conditions, tyrosinase functions to synthesize melanin in melanosomes to a specific level and the melanin produced is transferred to keratinocytes, which maintains the constitutive normal skin color. Thus, because of the difficulty of topically applying agents to a limited hyperpigmented skin area, potent anti-melanogenic agents capable of suppressing constitutive pigmentation may lead to hypopigmentation, which hampers further research into potent anti-melanogenic agents without that risk. From this viewpoint, abrogating the activation of melanogenesis, including the relevant melanogenic intracellular signaling pathways, would be an appropriate approach to develop new potent anti-pigmenting agents without the risk of hypopigmentation. In line with this approach, we have been looking for unique substances that have the potential to abolish the endothelin (EDN)-1 or stem cell factor (SCF)-stimulated pigmentation of human epidermal equivalents (HEEs). These anti-pigmenting effects should not be due to the inhibition of post-translational events, such as tyrosinase activity, but should interrupt intracellular linkages upstream of the expression of genes encoding melanocyte-specific proteins, such as tyrosinase and premelanosome protein (PMEL) 17. In this article, we review the hyperpigmentation mechanisms and intracellular signaling cascades that lead to the stimulation of melanogenesis and discuss perspectives of anti-pigmenting agents on the basis of our recent studies.

## 2. Paracrine Cytokine Mechanisms Underlying the Hyperpigmentation of UVB-Melanosis, Solar Lentigo and Melasma

**Figure 1.**
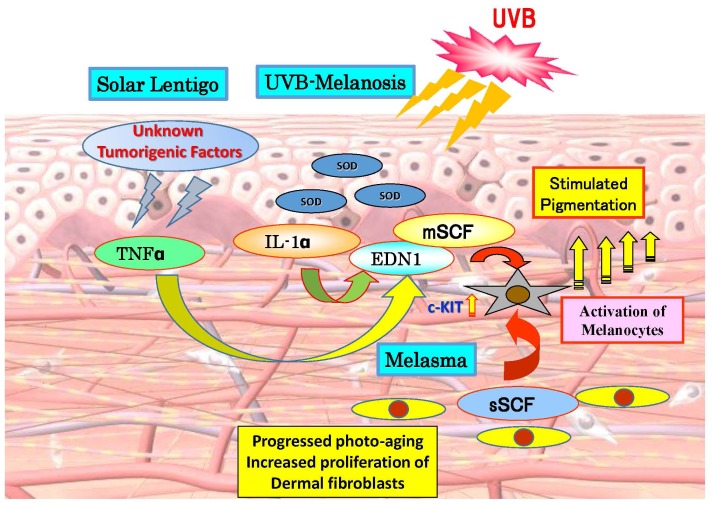
Paracrine cytokine mechanisms underlying hyperpigmentation in UVB-melanosis, solar lentigo and melasma. TNF, tumor necrosis factor; IL-1, interleukin-1; EDN1, endothelin-1; mSCF, membrane-bound stem cell factor; sSCF, soluble stem cell factor.

Hyperpigmentary disorders that appear especially on the face and are targeted by anti-pigmenting agents, include UVB-melanosis, solar lentigo and melasma. The hyperpigmentation mechanisms of those conditions have already been unraveled in detail, as depicted in Figure 1. In UVB-melanosis [4–11] and in solar lentigo [12,13], EDN1 and membrane-bound SCF (mSCF) are up-regulated at the production and/or secretion levels due to the UVB-stimulated release of interleukin (IL)-1 and the UV-independent secretion of tumor necrosis factor α (TNFα), respectively. Those cytokines cause neighboring melanocytes to stimulate their production of the critical melanin synthesizing enzyme, tyrosinase, via corresponding specific intracellular signaling cascades which are initiated and activated after the binding of EDN1 or SCF to the endothelin B receptor (EDNRB) or mast/stem cell growth factor receptor known as proto-oncogene *c-Kit* (*c-KIT*) respectively. In melasma [14], the secretion of soluble SCF (sSCF) by dermal fibroblasts [15] is up-regulated in the lesional dermis, probably due at least in part to the photoaging process [14]. This leads to the penetration of sSCF into the epidermis through the basement membrane and then to the activation of epidermal melanocytes via the SCF signaling cascade, resulting in the stimulation of epidermal pigmentation.

On the other hand, alpha-melanocyte stimulating hormone (αMSH) and adrenocorticotropic hormone (ACTH) have also been reported to be up-regulated both at the protein and mRNA levels upon treatment with UVB in human keratinocytes [16,17]. αMSH and ACTH have a distinct potential to stimulate human melanocytes to proliferate and synthesize melanin at concentrations above 100 nM in the presence of basic fibroblast growth factor (bFGF) [18,19]. However, those concentrations are relatively higher than physiologically effective concentrations compared with the significant effects of EDN1 [4,6] and SCF [9] at concentrations of 1–10 nM. According to criteria to determine if a cytokine is an intrinsic factor involved in UVB-hyperpigmentation (Table 1), the biological and physiological properties of αMSH do not fulfill those criteria, which suggests that αMSH is not involved in UVB-hyperpigmentation. Further, in contrast to intrinsic melanogenic cytokines such as EDN1 and SCF, there are no known hyperpigmentary disorders in which αMSH is an intrinsic melanogenic cytokine. Thus, as shown in Table 1, evidence as to whether the hyperpigmentation can be suppressed *in vivo*, either by antibodies that block the corresponding receptor(s) or by receptor antagonists, is the most essential requirement to determine if a cytokine is an intrinsic factor involved in UVB hyperpigmentation. In this respect, EDN1 and SCF have been identified as intrinsic melanogenic cytokines on the basis of evidence that UVB-induced hyperpigmentation or solar lentigo are ameliorated or diminished under *in vivo* conditions by substances (*M. chamomilla* extract) capable of interrupting the EDN1-signaling cascade [8,20] or by a blocking antibody (ACK2 monoclonal antibody) to *c-KIT* [9], respectively. In contrast, αMSH has not been identified as an intrinsic melanogenic cytokine in UVB-melanosis based on the following evidence: on the tail skin of C57BL/6J mice-aa/ee (recessive yellow), which have a mutation in the melanocortin 1 receptor (MC1R) and do not respond to MSH, UVB irradiation induced a distinct hyperpigmentation concomitant with an increased number of epidermal melanocytes and an increased activity of tyrosinase [21]. The pattern of the UVB-inducible pigmentation in the tail skin of recessive yellow mice was similar to the response of AA/EE (black), AA/eE (black) and A^y^e/EE (lethal yellow) mice which have wild-type functional MC1R [21]. Those findings strongly suggest that the αMSH triggered signaling pathway is not a major linkage for eliciting UVB-stimulated epidermal pigmentation. Further, while conditioned medium obtained from UVB-exposed human keratinocytes has a distinct potential to stimulate tyrosinase activity or DNA synthesis in human melanocytes, a neutralizing antibody to αMSH failed to abolish the stimulated tyrosinase activity when added to the conditioned medium. In that study, there was no detectable level of αMSH in the UVB-conditioned medium [22]. In contrast, in similar studies, EDN1 was found to be secreted into UVB-conditioned medium at physiological concentrations and the tyrosinase activity and DNA synthesis stimulated by the UVB-conditioned medium was significantly abrogated by the addition of a neutralizing antibody to EDN1 [22]. Such a failure to detect the secretion of αMSH at physiological concentrations by ELISA is consistent with the concentration of 31 pg/mL/0.5 × 10^6^ cells (which is equivalent to 18.62 pM) [16] at which αMSH is detectable only by radioimmunoassay in the conditioned medium of UVB-exposed normal human keratinocytes. The sum of these findings indicates that although αMSH is up-regulated at the transcriptional level in UVB-exposed epidermis, as is bFGF, it is not mechanistically involved in the stimulation of human epidermal pigmentation.

Based on the evidence that EDN1 and SCF are only the intrinsic melanogenic cytokines in hyperpigmentary disorders on the face being targeted by anti-pigmenting agents, it is strongly anticipated that substances able to interrupt the EDN1- or SCF-specific intracellular signaling pathways but do not directly inhibit tyrosinase activity, would be effective new anti-pigmenting agents. They would have the advantage that they do not affect normally pigmented skin where the intracellular signaling cascade is not activated, but would have distinct inhibitory effects on hyperpigmented areas of the skin with UVB-melanosis, solar lentigo and melasma. For such anti-pigmenting agents, there seems to be a low risk of eliciting hypopigmentation because they have no direct inhibitory effect on tyrosinase activity and because there is no activated intracellular signaling cascade in normally pigmented skin.

## 3. Intracellular Signaling Mechanisms Associated with Endothelin-1 (EDN1) and Stem Cell Factor (SCF)

**Figure 2.**
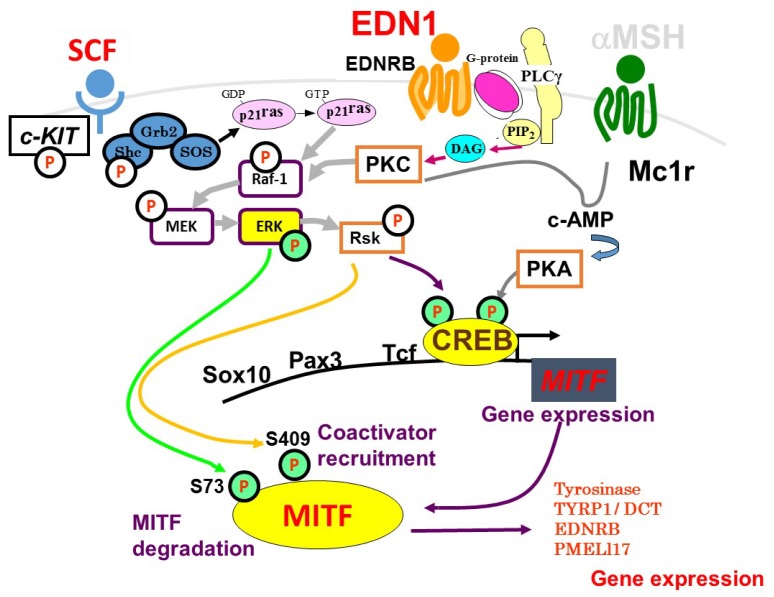
Intracellular signaling mechanisms associated with EDN1 and SCF. EDN1, endothelin-1; EDNRB, endothelin B receptor; MITF, microphthalmia associated transcription factor; PKA, protein kinase A; PKC, protein kinase C; SCF, stem cell factor; TYRP-1, tyrosinase-related protein-1; DCT, dopachrome tautomerase; TYK, tyrosine kinase; αMSH, alpha melanocyte stimulating hormone.

In NHMs, the intracellular signaling mechanisms that lead to melanogenic activation by ligands such as EDN1 and SCF have been well elucidated, as depicted in Figure 2. After binding to its receptor EDNRB, EDN1 triggers the hydrolysis of polyphosphoinositide, which generates inositol triphosphate (IP3) and diacylglycerol by the action of activated phospholipase Cγ, which mobilizes intracellular Ca^2+^ and activates PKC, respectively [4,7]. The activated PKC directly phosphorylates RAF proto-oncogene serine/threonine-protein kinase (Raf-1), at many serine residues, or the Raf-1 inhibitory protein that activates Raf-1 via complex mechanisms [23–26]. That then leads to the phosphorylation (activation) of a series of mitogen-activated protein kinase kinase (MEK)/extracellular signal-regulated mitogen-activated protein kinase (ERK1/2)/ribosomal s6 kinase (RSK)/cAMP response element binding protein (CREB) signaling molecules in the mitogen-activated protein kinase (MAPK) cascade during which the protein phosphorylation is mediated by the kinase activities of Raf-1 for MEK, of MEK for ERK, of ERK for RSK, of RSK for CREB and of ERK/RSK for MITF [27,28]. The activation of PKC also results in increased levels of cyclic AMP and the subsequent activation of PKA elicits the phosphorylation of CREB, which is also induced by the action of RSK following ERK activation [7,28].

On the other hand, SCF, which binds to the *c-KIT* receptor, mediates the activation of its intrinsic tyrosine kinase activity via dimerization and subsequent autophosphorylation [29]. The activated *c-KIT* receptor then phosphorylates various substrates and associates with a number of different signaling molecules, including the SH2, Src homology domain (Shc) and growth factor receptor binding protein 2 (Grb2) adaptor proteins, and the guanine nucleotide exchange factor, SOS all of which lead to the conversion from Rat sarcoma-guanosine diphosphate (Ras-GDP) to Rat sarcoma-guanosine triphosphate (Ras-GTP) [30–32]. The phosphorylation of Raf-1 is mediated by Ras-GTP and the activation of Raf-1 then leads to phosphorylation (activation) of the series of MEK/ERK1/2/RSK/CREB signaling molecules in the MAPK cascade as detailed above. Thus, between EDN1 and SCF signaling in NHMs, the intracellular signaling pathways consisting of Raf-1/MEK/ERK/MITF/CREB overlap with each other. The activation of CREB through dual phosphorylation by both PKA and RSK activations in EDN1 signaling and a phosphorylation by RSK activation in SCF signaling results in increased gene and protein expression of the melanocyte-master transcription factor, MITF [28]. While being phosphorylated by ERK and RSK kinases, increased levels of phosphorylated and non-phosphorylated MITF in turn lead, in combination with other transcription factors, such as SOX10, PAX3, lymphoid-enhancing factor-1 (LEF-1) and T cell factor (TCF) [33,34], to up-regulated gene and protein expression levels of several melanocyte-specific proteins. Those include tyrosinase [35], tyrosinase-related protein-1 (TYRP1), dopachrome tautomerase (DCT) [36], PMEL17 [37], EDNRB [28] and *c-KIT* [38], all of which contribute to the stimulation of epidermal pigmentation.

We have already found that there is a mutual interaction in intracellular signaling between EDN1 and SCF, which synergistically stimulates DNA synthesis and melanogenesis via cross-talk between EDN1-induced PKC activation and SCF-induced *c-KIT* auto-phosphorylation (activation). That initiates synergistic *c-KIT* activation and leads to synergistic activation through the *c-KIT*/Shc/Raf-1/MEK/ERK/CREB/MITF linkage in NHMs [27]. Among several melanogenic cytokines (bFGF, GM-CSF, αMSH, HGF, growth-related oncogene α (GROα), such a synergistic interaction occurs only between EDN1 and SCF [7]. Since the hyperpigmentation in UVB-melanosis and solar lentigo occurs distinctly based on the synergistic stimulation between EDN1 and SCF, this suggests that interruption of either one or both of those intracellular signaling cascades would abolish the synergistic cross-talk signaling, resulting in a distinct anti-pigmenting effect.

## 4. Effects of Specific Signaling Inhibitors on EDN1 or SCF Signaling

Before screening for inhibitors of melanogenic intracellular signaling, we asked if specific signaling inhibitors can interrupt the EDN1- or SCF-induced activation of signaling molecules in acral lentigo malignant (ALM) melanoma cells, which respond to melanogenic ligands in a fashion similar to NHMs [39,40]. Among the specific EDN1 signaling inhibitors tested (PB98059, H89 and Gö6983), the MEK inhibitor, PD98059, significantly suppressed the EDN1-stimulated phosphorylation of ERK and of MITF at 5–30 min post-treatment in EDN1-treated ALM melanoma cells, whereas it only slightly reduced the phosphorylation of CREB [39]. Similarly, a PKC inhibitor, Gö6983, significantly suppressed the EDN1-stimulated phosphorylation of ERK, MITF and CREB at 5–30 min post-EDN1 treatment [39]. In contrast, a PKA inhibitor, H89, did not affect the EDN1-stimulated phosphorylation of ERK, MITF or CREB at 5–30 min post-EDN1 treatment [39].

**Figure 3.**
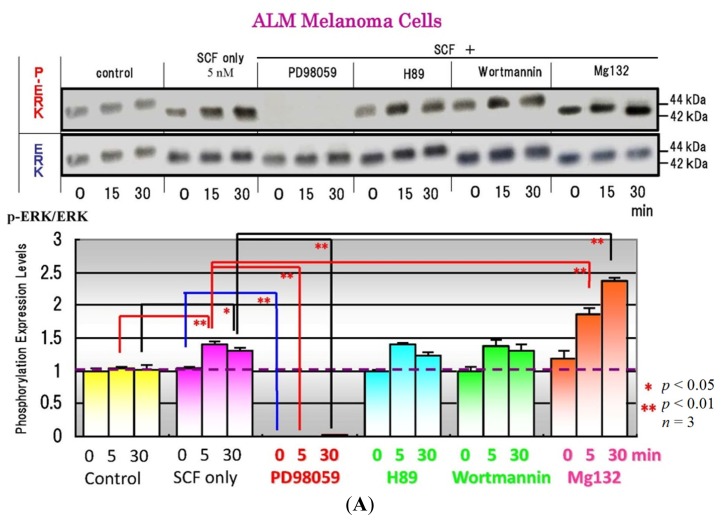

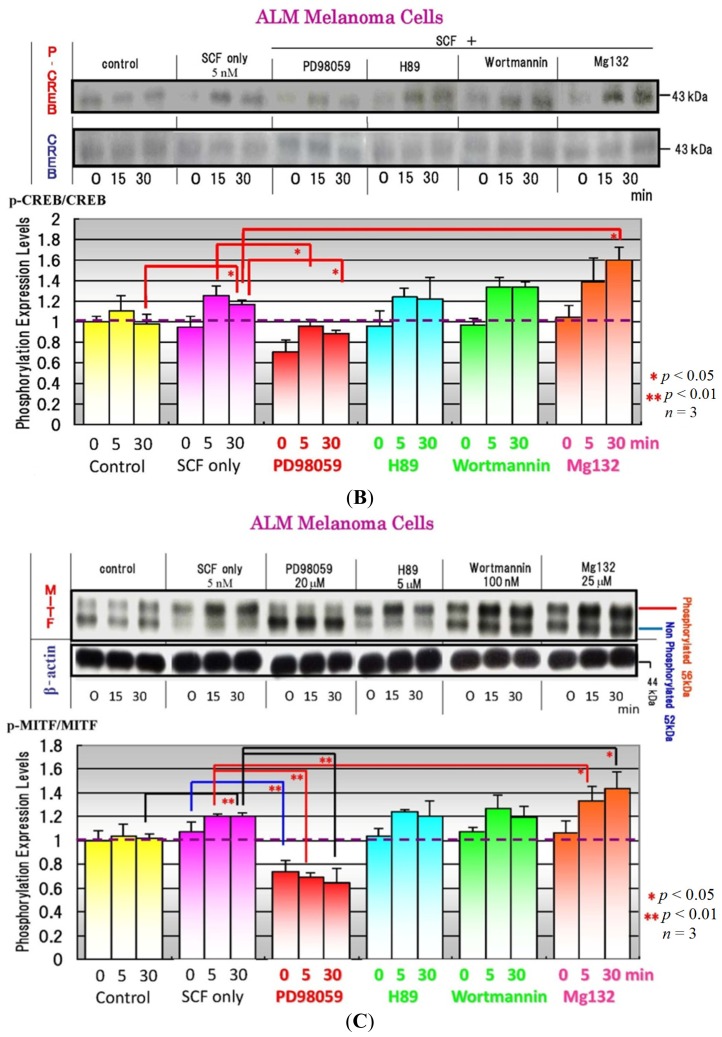
Effects of specific signaling inhibitors on ERK (**A**); CREB (**B**); MITF (**C**) phosphorylation during SCF signaling.

Among the specific SCF signaling inhibitors tested (PB98059, H89 and *Wortmannin*), the MEK inhibitor, PD98059, significantly suppressed the SCF-stimulated phosphorylation of ERK and of MITF at 5–30 min post-treatment in SCF-treated ALM melanoma cells, whereas H89 and *Wortmannin* did not affect the SCF-stimulated phosphorylation of ERK, MITF or CREB during that time (Figure 3). On the other hand, addition of the proteasome inhibitor MG132 significantly stimulated the phosphorylation of ERK, CREB and MITF, probably due to the abrogation of their quick degradation (Figure 3).

## 5. Effects of EDN1 or SCF on the Pigmentation of Human Epidermal Equivalents (HEEs)

**Figure 4.**
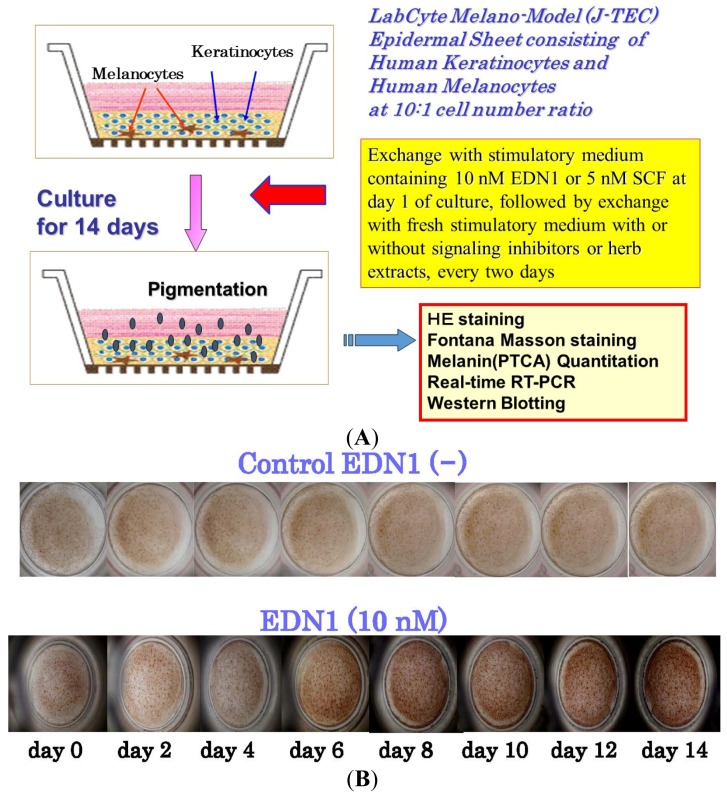

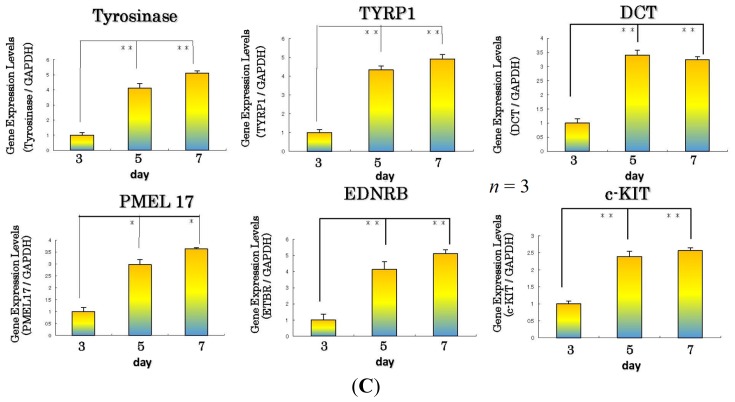
(**A**) Experimental procedure for human epidermal equivalents (HEEs) using the J-TEC Melano-Model [39–42]; (**B**) Effects of EDN1 on the pigmentation of HEEs during 14 days of culture; (**C**) Time course of expression of melanocyte-specific genes in the EDN1-stimulated pigmentation of HEEs. * *p* < 0.05; ** *p* < 0.01. EDN1, endothelin-1; SCF, stem cell factor, PTCA, pyrrole-2,3,5-tricarboxylic acid.

To develop a model for epidermal hyperpigmentary disorders that are mechanistically associated with EDN1 or SCF as intrinsic melanogenic cytokines, we used HEEs to examine the stimulatory effects of EDN1 or SCF on epidermal pigmentation [38,40–42] (Figure 4A). The addition of EDN1 gradually stimulated the visible pigmentation of HEEs over 14 days of treatment (Figure 4B). A time course study using real-time polymerase chain reaction (RT-PCR) demonstrated that the expression levels of all genes encoding melanocyte-specific proteins (tyrosinase, TYRP1, DCT, PMEL17, EDNRB and *c-KIT*) tested were gradually up-regulated over 10 days of EDN1 treatment, with a peak at days 7–10 (Figure 4C). Thus, we succeeded in establishing an EDN1-associated pigmentation stimulation model with HEEs, which mimics the hyperpigmentation observed in UVB melanosis and solar lentigo. In those same HEEs, the addition of SCF also gradually stimulated the visible pigmentation over 14 days of treatment [42]. A time course study using RT-PCR and western blotting demonstrated that the expression levels of all melanocyte-specific genes and proteins (Tyrosinase, TYRP1, DCT, PMEL17, EDNRB and *c-KIT*) tested were gradually up-regulated over 10 days of SCF treatment with a peak at days 5 and 7 for MITF or at days 7–10 and 10–12 for the others [42].

## 6. Effects of Signaling Inhibitors on EDN1 or SCF-Stimulated Pigmentation

**Figure 5.**
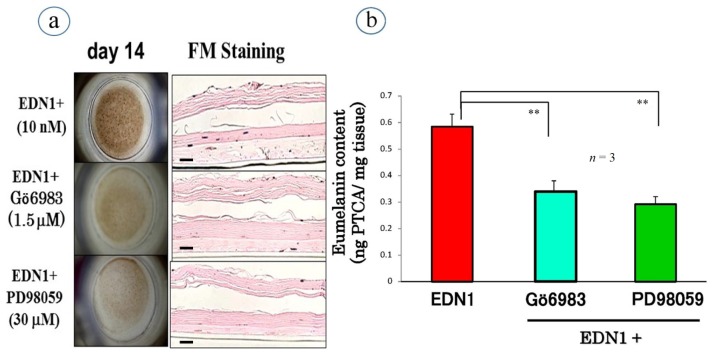
Inhibitory effects of signaling inhibitors on the EDN1-stimulated pigmentation/Hematoxylin and eosin stain (HE)/Fontana Masson (FM) staining (**a**); eumelanin content (PTCA) (**b**) of HEEs at day 14. ** *p* < 0.01; Bar = 100 μm.

Before associating the depigmenting effects of selected signaling inhibitors with the interruption of the EDN1 or SCF signaling cascades, we asked if a PKC, Phosphoinositide (PI) 3-kinase or MEK inhibitor can affect the EDN1 or SCF-stimulated pigmentation of HEEs [39,42]. During the EDN1-stimulated pigmentation, a PKC or MEK inhibitor markedly abolished the visible pigmentation over 14 days (Figure 5a). While there was no degeneration of the epidermal tissue in the signaling inhibitor-treated HEEs at day 14, melanin deposition throughout the epidermis was significantly reduced in the PKC or MEK inhibitor-treated HEEs compared with the untreated control (Figure 5a). HPLC analysis of the eumelanin content in HEEs revealed that the PKC and MEK inhibitors significantly abrogated the eumelanin content (pyrrole-2,3,5-tricarboxylic acid (PTCA) ng/mg tissue) compared with the untreated control (Figure 5b).

We next determined whether a MEK inhibitor or the PI3 kinase inhibitor *Wortmannin* affected the SCF-stimulated pigmentation of HEEs [42]. During the SCF-stimulated pigmentation, the MEK inhibitor PD90859 markedly abolished the visible pigmentation of HEEs over 14 days whereas the PI3 kinase inhibitor *Wortmannin* did not. While there was no degeneration of the epidermal tissue in the MEK inhibitor-treated HEEs at day 14, increased melanin deposition throughout the epidermis was markedly abrogated in the MEK inhibitor (PD98059)-treated HEEs compared with the untreated control. In contrast, the PI3 inhibitor-treated HEEs had levels of melanin deposition similar to the untreated control. HPLC analysis of HEEs revealed that the MEK inhibitor significantly abrogated the increased level of eumelanin content (PTCA ng/mg tissue) compared with the untreated control. In contrast, the PI3 inhibitor *Wortmannin* did not affect the eumelanin content.

## 7. Screening of Herb Extracts or Natural Chemicals Capable of Interrupting the EDN1 or SCF-Triggered Intracellular Signaling Cascades

**Figure 6.**
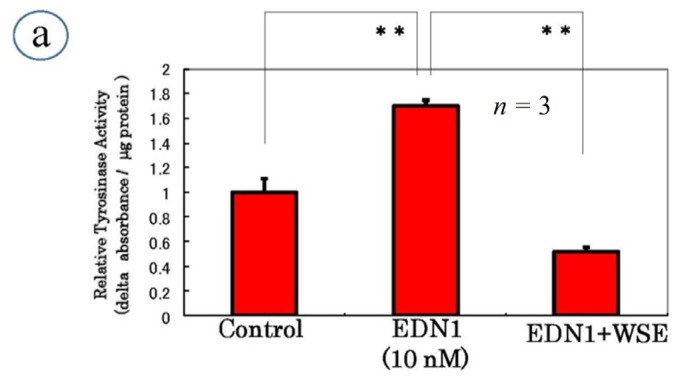

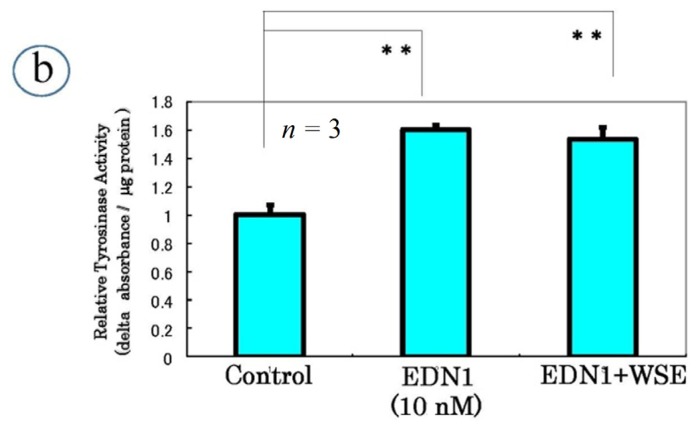
Effects of the *Withania somnifera* extract (WSE) on tyrosinase activity. (**a**) Addition 3 h before EDN1 stimulation; (**b**) Direct addition to cell lysate 72 h after EDN1 stimulation, EDN1: 10 nM, WSE: 10 μg/mL. ** *p* < 0.01.

To search for inhibitors of EDN1 or SCF signaling, we used EDN1- or SCF-treated NHMs to test if the increased tyrosinase activity after 72 h is significantly abrogated by 3 h pre-incubation with a herb extract or natural chemical compared with the unstimulated basal level. When active substances were identified, lysates of NHMs cultured in the absence of the active substance(s) 72 h after SCF or EDN1 stimulation were directly incubated with the active substance(s) after which tyrosinase activity was measured to test if the substance(s) served as a direct inhibitor of tyrosinase activity. Substances with no direct inhibitory effect on tyrosinase activity were subjected to further evaluation. As a result of the screening of many kinds of herb extracts, we identified the *Withania somnifera* extract (WSE) as a candidate for an inhibitor of the EDN1-triggered intracellular signaling cascade [41]. The results showed that tyrosinase activity in lysates of NHMs cultured for 72 h was significantly suppressed by 3 h pre-incubation with the WSE before EDN1 stimulation that did not exceed the unstimulated basal level (Figure 6). In contrast, tyrosinase activity was not affected by the direct addition of the WSE (Figure 6). These findings indicate there is no direct inhibition of tyrosinase activity by the WSE and that the WSE interrupts an upstream pathway that leads to tyrosinase expression. At the effective concentrations of the WSE, there was no cytotoxic change on NHM morphology and the WSE-treated NHMs were completely viable [41].

## 8. Withania somnifera

*Withania somnifera*, commonly known as *Ashwagandha* or Indian winter cherry, is a medicinal plant that contains *Withaferin* A (WFA) as a bioactive steroidal lactone. The known pharmacologic effects of *WSE* are associated with the modulation of immune function [41–43], cardioprotection from ischemia and reperfusion injury [44], protection of 6-hydroxydopamine–induced Parkinsonism in rats [45], antibacterial effects [46] and anti-inflammatory effects [47]. As for its effects modulating signal transduction, cell signaling, transcription, apoptosis and the cell cycle, the WSE has been shown to suppress the lipopolysaccharide-induced production of inflammatory cytokines, including tumor necrosis factor (TNF) α, interleukin (IL)-1 and IL-12, by peripheral blood mononuclear cells [48], and it potently inhibits nuclear factor-NFκB activation [49,50]. The WSE also significantly down-regulates the expression of proinflammatory cytokines (IL-6, IL-1β, IL-8, Hsp70 and STAT-2) in prostate cancer cells, while a reciprocal up-regulation occurs in the expression of p38 MAPK, PI3K, caspase 6, cyclin D and c-myc [51]. Furthermore, the WSE significantly modulates the Janus kinase (JAK) and signal transducer activator of transcription (STAT) pathway, which regulates both the apoptosis process and MAPK signaling [51].

## 9. Effect of the *Withania somnifera* Extract (WSE) on EDN1 Signaling in Acral Lentigo Malignant (ALM) Melanoma Cells

**Figure 7.**
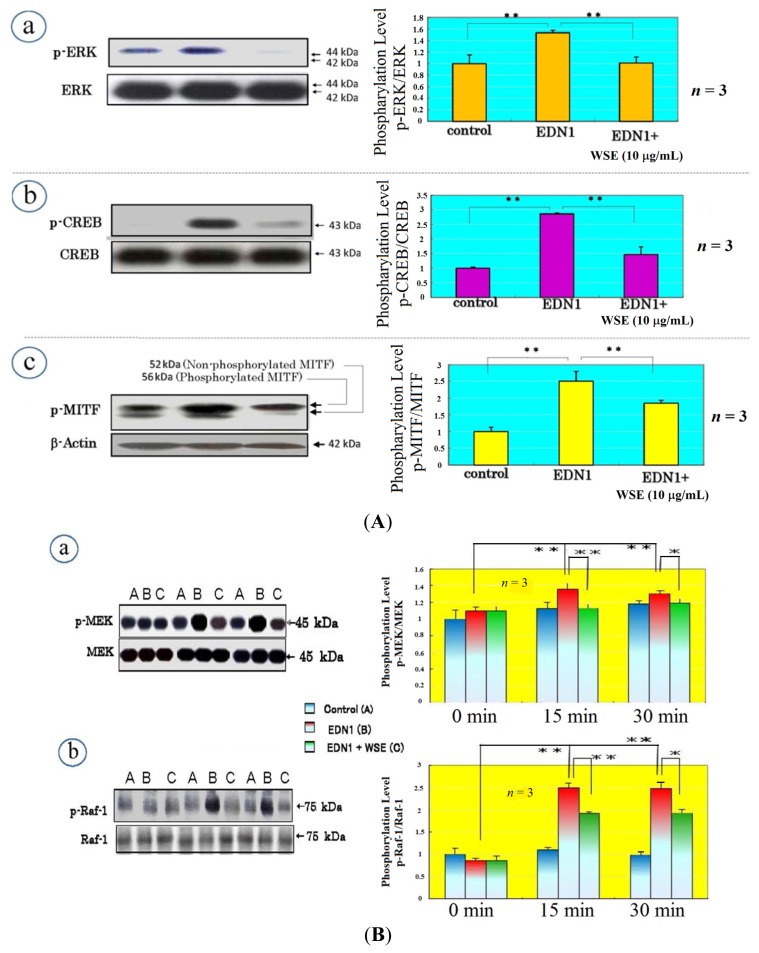

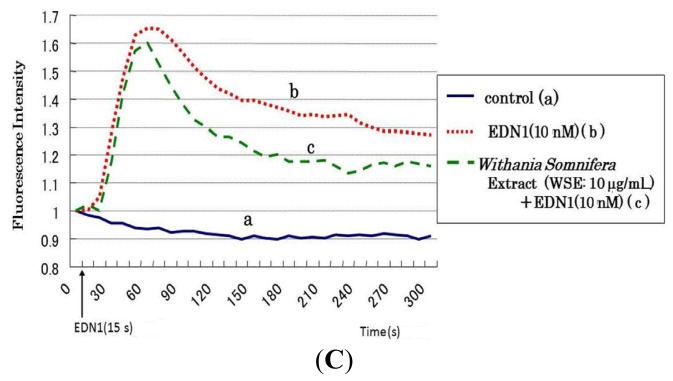
(**A**) Effect of the WSE on the EDN1-stimulated phosphorylation of ERK (**a**), CREB (**b**) and MITF (**c**) in ALM melanoma cells; (**B**) Effect of the WSE on the EDN1-stimulated phosphorylation of MEK (**a**) and Raf-1 (**b**) in ALM melanoma cells; (**C**) Effect of the WSE on the EDN1-induced mobilization of intracellular calcium in NHMs, (a) control; (b) EDN1 (10 nM); (c) WSE 10 mg/mL + EDN1 (10 nM). * *p* < 0.05; ** *p* < 0.01.

**Figure 8.**
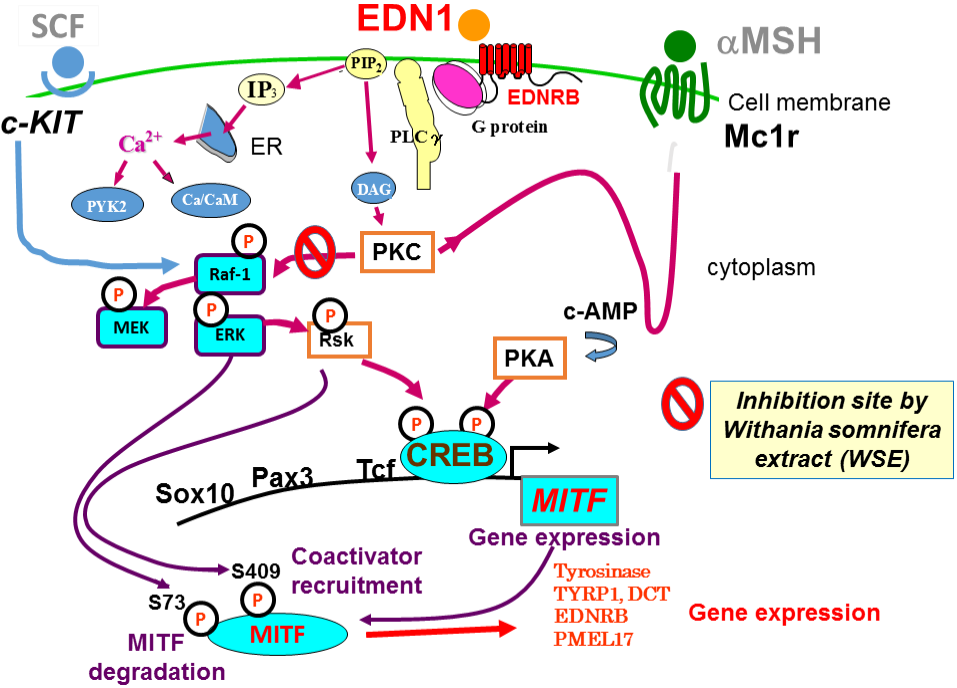
EDN1-activated intracellular signaling pathways leading to the expression of melanocyte-specific proteins and the signaling step affected by the WSE.

We next asked if the WSE interrupts the EDN1-induced phosphorylation of ERK, CREB and/or MITF. When the WSE was added 3 h prior to EDN1 stimulation, the EDN1-stimulated phosphorylation of ERK, CREB and MITF was significantly suppressed at 15 min post-treatment (Figure 7A). To determine which signaling factor(s) through the MAPK pathway is affected by the WSE, we examined its effects on the phosphorylation of MEK and Raf-1, which occur upstream of ERK, at 15–30 min post-treatment. Western blotting analysis revealed that the WSE significantly suppressed MEK and Raf-1 phosphorylation at 15–30 min after EDN1 incubation (Figure 7B). This result indicates that the WSE interrupts a signaling pathway upstream of Raf-1 activation, which results in the down-regulated phosphorylation of Raf-1 and MEK. To determine which signaling factor(s) upstream of Raf-1 is affected by the WSE, we examined their effects on the calcium mobilization which occurs upstream of PKC at 0–300 s after EDN1 incubation. Calcium mobilization analysis revealed that the WSE does not suppress the EDN1-induced mobilization of calcium starting at 15 s after EDN1 incubation (Figure 7C). This result indicates that the WSE abolishes PKC activation or inhibits its activity, resulting in the down-regulated phosphorylation of Raf-1, as depicted in Figure 8.

## 10. Effect of the WSE on the EDN1-Stimulated Pigmentation of HEEs

**Figure 9.**
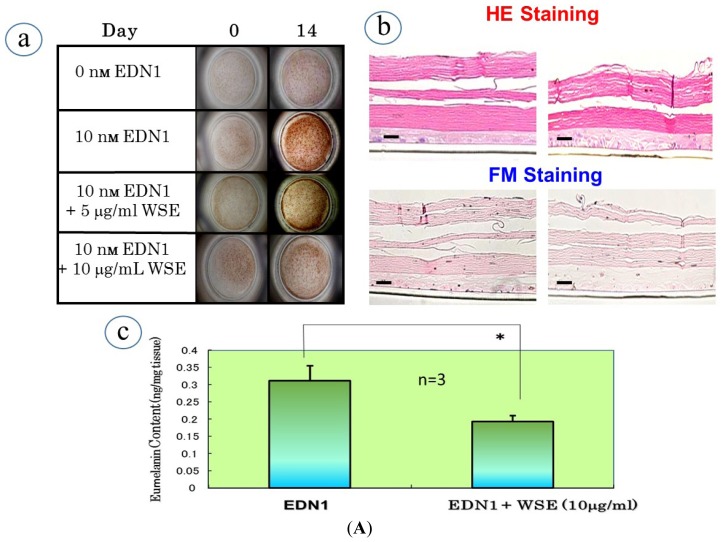

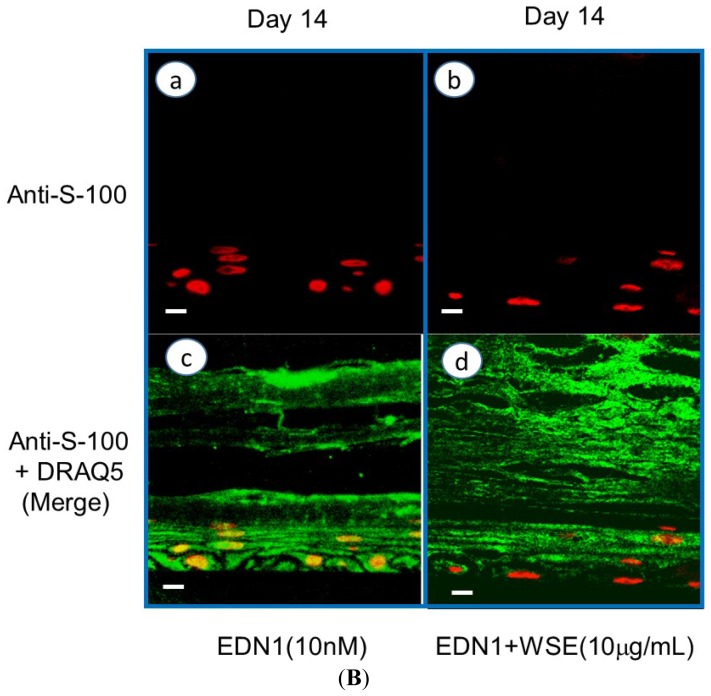
(**A**) Inhibitory effect of the WSE on the EDN1-stimulated pigmentation of HEEs: (**a**) Pigmentation; (**b**) HE staining and Fontana Masson (FM) staining of WSE-treated HEEs at day 14; Bar = 100 μm; (**c**) HPLC analysis of eumelanin content in WSE-treated HEEs at day 14; (**B**) Immunohistochemistry of S-100 staining at day 14: (**a**) Immunostaining with anti-S-100 following treatment with EDN1 (10 nM); (**b**) Immunostaining with anti-S-100 following treatment with EDN1 (10 nM) + WSE (10 μg/mL); (**c**) Immunostaining with anti-S-100 + DRAQ5 following treatment with EDN1 (10 nM); (**d**) Immunostaining with anti-S-100 + DRAQ5 following treatment with EDN1 (10 nM) + WSE (10 μg/mL). * *p* < 0.05; Bar = 100 μm.

It has become evident that the WSE has a distinct potential to interrupt the EDN1-activated intracellular signaling cascade during which ERK/CREB phosphorylation (activation) leading to the up-regulation of MITF is distinctly attenuated by pre-incubation with the extract. Since the inhibition by a MEK or PKC inhibitor of ERK/CREB phosphorylation elicited by EDN1 stimulation attenuates the EDN1-stimulated pigmentation of HEEs, we asked if the WSE elicits an abrogating effect on the EDN1-stimulated pigmentation in HEEs. During the EDN1-induced stimulation of pigmentation, the addition of the WSE at concentrations of 5 or 10 μg/mL distinctly reduced the increase in visible pigmentation in a dose-dependent manner over those 14 days with the most marked suppression attained at 10 μg/mL (Figure 9A). Those suppression levels were slightly higher than the basal pigmentation levels without EDN1 stimulation. While there was no degeneration of the epidermal tissue visible at day 14 (detected by hematoxylin and eosin (HE) staining), melanin deposition (detected by Fontana Masson staining) throughout the epidermis was markedly reduced in the WSE-treated HEEs at day 14 compared to the untreated controls (Figure 9A). Chemical analysis revealed that the WSE significantly reduced the eumelanin content (ng PTCA/mg tissue) compared with the untreated control (Figure 9A). Immunohistochemistry revealed that there was no reduction in the number of S-100 positive melanocytes in the WSE-treated HEEs at day 14 compared with the untreated controls and the non-EDN1 stimulation control (Figure 9B). The lack of a reduced number of S-100 positive melanocytes strongly suggests that there is no melano-cytotoxic effect in the depigmenting effect elicited by the WSE.

## 11. Effect of the WSE on the Expression of Melanocyte-Specific Genes and Proteins

**Figure 10.**
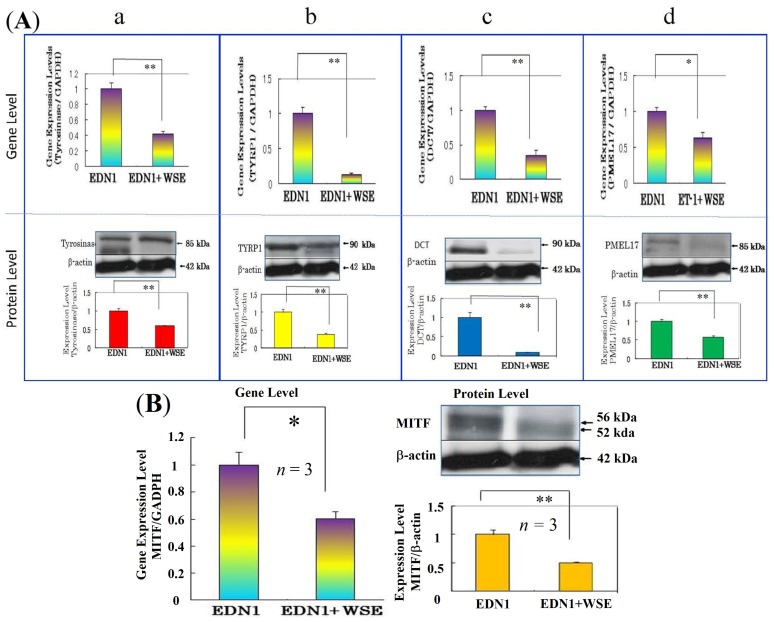
(**A**) Inhibitory effects of the WSE on EDN1-stimulated gene and protein expression at days 7 and 10, respectively: (**a**) Tyrosinase; (**b**) TYRP1; (**c**) DCT; (**d**) PMEL17; *n* = 3; (**B**) Inhibitory effects of the WSE on EDN1-stimulated gene and protein expression of MITF at days 7 and 10, respectively. * *p* < 0.05; ** *p* < 0.01.

To elucidate the biochemical mechanism(s) involved in the anti-pigmenting effect of the WSE, we used RT-PCR analysis to examine its effects on the expression of melanocyte-specific genes during the EDN1-stimulated pigmentation of HEEs. A time course study demonstrated that the expression of the melanocyte-specific genes tested (Tyrosinase, TYRP1, DCT and PMEL17) was gradually up-regulated over 14 days with a peak at day 7. RT-PCR analysis of the effects of the WSE at day 7 revealed that the addition of the WSE at a concentration of 10 μg/mL significantly down-regulated the increased expression of those melanogenic genes compared with the untreated controls (Figure 10A). To further elucidate the biochemical mechanisms involved in the anti-pigmenting effect of the WSE, we next used Western blotting analysis to examine its effects on the levels of melanocyte-specific proteins at day 10, at which time the protein expression has peaked during the EDN1-stimulated pigmentation of HEEs. Western blotting analysis revealed that addition of the WSE significantly abolished the increased levels of all melanocyte-specific proteins examined compared with the untreated control at day 10 (Figure 10A). The down-regulated expression of these melanocyte-specific genes and proteins by the *WSE* was accompanied by the similar down-regulated expression of MITF (Figure 10B), which suggests that the abrogating effect on the up-regulated expression of the melanocyte-specific genes and proteins is mainly attributable to the suppressive effect on the EDN1-enhanced expression of MITF.

## 12. Summary of Substances Identified that Are Capable of Interrupting the SCF- or EDN1-Activated Intracellular Signaling Cascades and Their Abrogating Effects on the SCF- or EDN1-Stimulated Pigmentation in HEEs

In addition to the *WSE* as an inhibitor of EDN1-triggerred intracellular signaling, as shown in Table 2, we have found that there are other herb extracts and natural chemicals with an abrogating effect on the SCF- or EDN1-stimulated activity of tyrosinase as well as an interrupting effect on SCF- or EDN1-triggered intracellular signaling. Interestingly, the WSE also inhibits SCF-triggered intracellular signaling and elicits an abrogating effect on the SCF-stimulated pigmentation in HEEs [40]. In contrast, a *Melia toosendan* extract (MTE) has a distinct potential to interrupt the EDN1-triggered intracellular signaling cascade and also to abrogate the EDN-stimulated pigmentation of HEEs. The MTE fails to interrupt the SCF-triggered intracellular signaling cascade and consistently does not abrogate the SCF-stimulated pigmentation of HEEs [39]. Using the same screening system, we also found that the natural chemical astaxanthin has a distinct potential to interrupt the SCF-triggered intracellular signaling cascade and to abrogate the SCF-stimulated pigmentation of HEEs. However, astaxanthin fails to interrupt the EDN1-triggered intracellular signaling cascade and consistently is not able to abrogate the EDN1-stimulated pigmentation of HEEs [42]. Further, *Withaferin* A, one of the active chemicals in the WSE, was found to exhibit a distinct potential to interrupt both the EDN1- and SCF-triggered intracellular signaling cascades and consistently abrogates both the EDN1- and SCF-stimulated pigmentation of HEEs [22,52].

## 13. Conclusions

Among the 4 types of herb extracts or natural chemicals discussed above, there is a consistency between the abilities to abrogate the up-regulated activity of tyrosinase and to interrupt the EDN1- or SCF-triggered intracellular signaling cascades and to abolish the EDN1- or SCF-stimulated pigmentation of HEEs. The sum of these findings suggests that inhibitors of EDN1- or SCF-triggered intracellular signaling are appropriate candidates for anti-pigmenting agents with a low risk of hypopigmentation and with excellent efficacy. Of course, when applied on the skin, anti-pigmenting agents are required to penetrate at effective concentrations into the epidermis where melanocytes are located in the bottom layers in order for them to exert their anti-melanogenic effects on melanocytes. In general, active chemicals included in herb extracts and natural chemicals tested have lower molecular weights and have lipophilic properties which facilitate their cutaneous permeability. Therefore, it is anticipated that inhibitors of SCF- or EDN1-triggered intracellular signaling cascades are appropriate anti-pigmenting agents capable of down-regulating the hyperpigmentation observed in UVB-melanosis, solar lentigo and melasma. It is desirable that substances capable of interrupting both the EDN1- and SCF-triggered intracellular signaling cascades are selected as candidates for new anti-pigmenting agents. However, in light of the synergistic stimulation in intracellular signaling between EDN1 and SCF, the interruption of either or both of those intracellular signaling cascades may abolish the synergistic cross-talk signaling, resulting in exerting a distinct anti-pigmenting effect on these hyperpigmentary disorders.

## Figures and Tables

**Table 1.** Criteria to determine if a cytokine is an intrinsic factor involved in UVB-hyperpigmentation.

Five criteria to determine if a cytokine is an intrinsic factor involved in UVB hyperpigmentation	EDN1	mSCF	αMSH	bFGF

	EDNRB	*c-KIT*	MC1R	*c-Met*
1. The cytokine(s) should have the potential to activate melanocytes at physiological concentration *in vitro*.	○ (1–10 nM)	○ (1–10 nM)	Δ (>100 nM)	Δ (>100 nM)
2. The cytokine(s) should exist in supernatants of UVB-exposed keratinocytes at concentrations sufficient to stimulate melanocytes.	○	–	○	×
3. The stimulatory effect of culture supernatants on melanocytes should be neutralized by an antibody to the cytokine if it is secretable.	○	–	○	×
4. The cytokine(s) should be highly expressed in UVB-exposed epidermis.	○	○	○	○
5. The hyperpigmentation induced should be suppressed by antibodies that inhibit the corresponding receptor or by receptor antagonists *in vivo*.	○	○	×	×

○, Conformable; ×, Not Conformable; Δ, Partly Conformable; EDN1, endothelin-1; EDNRB, endothelin B receptor; *c-KIT*, mast/stem cell growth factor receptor known as proto-oncogene *c-Kit*; αMSH, alpha melanocyte stimulating hormone; MC1R, melanocortin 1 receptor; bFGF, basic fibroblast growth factor; *c-Met*, Met proto-oncogene (hepatocyte growth factor receptor).

**Table 2.** Summary of substances capable of interrupting the SCF- or EDN1-activated intracellular signaling cascades and their abrogating effects on SCF- or EDN1-stimulated pigmentation of HEEs.

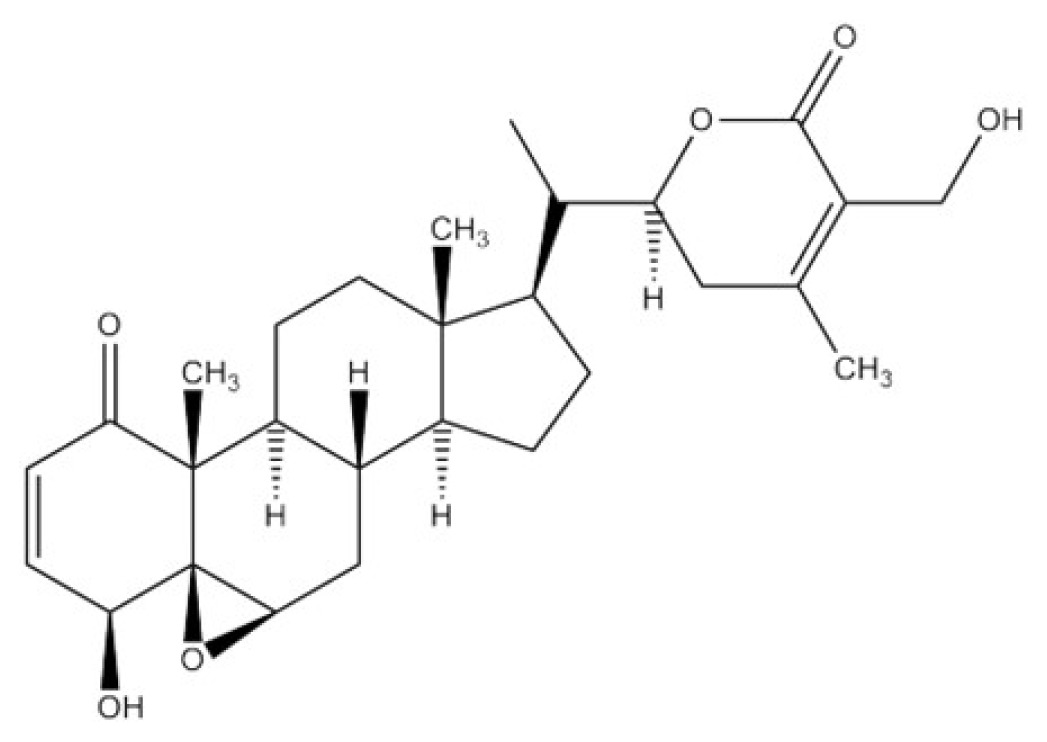 Withaferin A (WFA)				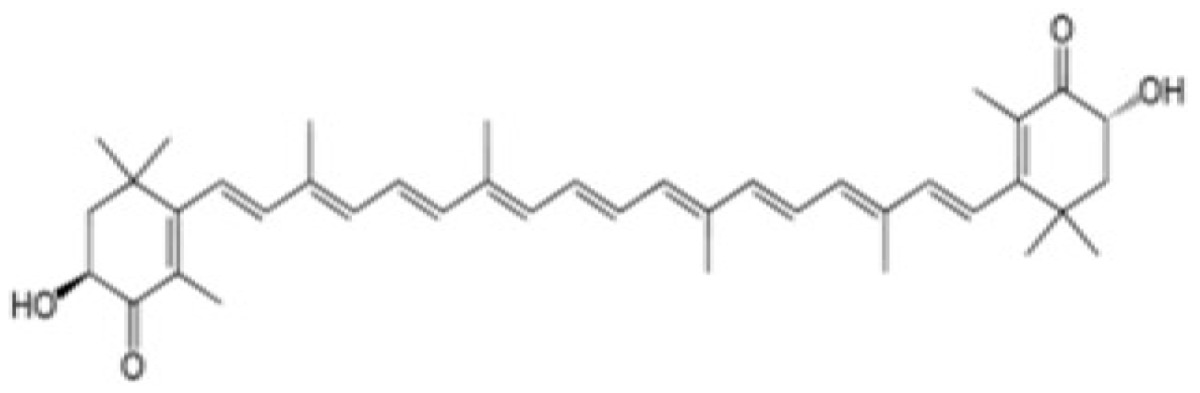 Astaxanthin (AX)				

Herb extract of natural chemical	Tyrosinase activity		Cytotoxicity (MTT assay in NHM)	Inhibitory effects on signaling and targeted signal molecules		3D human epidermal equivalent		Reference
	
	Direct inhibition	Abrogating effect		SCF signaling	EDN1 signaling	SCF	EDN1	
*Withania somnfera* (WSE)	○	×	○	Ras/Raf-1	PKC/Raf-1	×	×	[40,50]
*Melia toosendan* (MTE)	○	×	○	○	PKC/Raf-1	○	×	[39]
*Astaxanthin* (AX)	○	×	○	Raf-1/MEK	○	×	○	[51]
*Withaferin* A (WFA)	○	×	○	c-KIT	PKC/Raf-1	×	×	[22,39,52]

×, suppressive; ○, Not suppressive. The MTT assay is a colorimetric assay for assessing cell viability based upon the principle that NAD(P)H-dependent cellular oxidoreductase enzymes reflect the number of viable cells present, which are capable of reducing the tetrazolium dye MTT (3-(4,5-dimethylthiazol-2-yl)-2,5-diphenyltetrazolium bromide) to its insoluble formazan with a purple color.

## Abbreviations

EDN1: endothelin-1
EDNRB: endothelin B receptor
IL: interleukin
MAPK: mitogen activated protein kinase
MITF: microphthalmia associated transcription factor
NHM: normal human melanocytes
PKA: protein kinase A
PKC: protein kinase C
SCF: stem cell factor
TYRP-1: tyrosinase-related protein-1
DCT: dopachrome tautomerase
TYK: tyrosine kinase
WSE: *Withania somnifera* extract
MTE: *Melia toosendan* extract
TNF: tumor necrosis factor
αMSH: alpha melanocyte stimulating hormone
bFGF: basic fibroblast growth factor
ACTH: adrenocorticotropic hormone
MC1R: melanocortin 1 receptor
HEEs: human epidermal equivalents
PAR2: proteinase-activated receptor 2

## References

[1] Chung K.W., Park Y.J., Choi Y.J., Park M.H., Ha Y.M., Uehara Y., Yoon J.H., Chun P., Moon H.R., Chung H.Y. (2012). Evaluation of *in vitro* and *in vivo* anti-melanogenic activity of a newly synthesized strong tyrosinase inhibitor (*E*)-3-(2,4-dihydroxybenzylidene) pyrrolidine-2,5-dione (3-DBP). Biochim. Biophys. Acta.

[2] Ando H., Funasaka Y., Oka M., Ohashi A., Furumura M., Matsunaga J., Matsunaga N., Hearing V.J., Ichihashi M. (1999). Possible involvement of proteolytic degradation of tyrosinase in the regulatory effect of fatty acids on melanogenesis. J. Lipid Res.

[3] Paine C., Sharlow E., Liebel F., Eisinger M., Shapiro S., Seiberg M. (2001). An alternative approach to depigmentation by soybean extracts via inhibition of the PAR-2 pathway. J. Investig. Dermatol.

[4] Yada Y., Higuchi K., Imokawa G. (1991). Effects of endothelins on signal transduction and proliferation in human melanocytes. J. Biol. Chem.

[5] Imokawa G., Yada Y., Miyagishi M. (1992). Endothelins secreted from human keratinocytes are intrinsic mitogens for human melanocytes. J. Biol. Chem.

[6] Imokawa G., Miyagishi M., Yada Y. (1995). Endothelin-1 as a new melanogen: Coordinated expression of its gene and the *tyrosinase* gene in UVB-exposed human epidermis. J. Investig. Dermatol.

[7] Imokawa G., Yada Y., Kimura M. (1996). Signaling mechanisms of endothelin-induced mitogenesis and melanogenesis in human melanocytes. Biochem. J.

[8] Imokawa G., Kobayashi T., Miyagishi M., Higashi K., Yada Y. (1997). The role of endothelin-1 in epidermal hyperpigmentation and signaling mechanisms of mitogenesis and melanogenesis. Pigment Cell Res.

[9] Hachiya A., Kobayashi A., Ohuchi A., Takema Y., Imokawa G. (2001). The paracrine role of stem cell factor/c-kit signaling in the activation of human melanocytes in ultraviolet B-induced pigmentation. J. Investig. Dermatol.

[10] Hachiya A., Kobayashi T., Takema Y., Imokawa G. (2002). Biochemical characterization of endothelin-converting enzyme-1α in cultured skin-derived cells and its postulated role in the stimulation of melanogenesis in human epidermis. J. Biol. Chem.

[11] Hachiya A., Kobayashi A., Yoshida Y., Kitahara T., Takema Y., Imokawa G. (2004). Biphasic expression of two paracrine melanogenic cytokines, stem cell factor and endothelin-1, in ultraviolet B-induced human melanogenesis. Am. J. Pathol.

[12] Kadono S., Manaka I., Kawashima M., Kobayashi T., Imokawa G. (2001). The role of the epidermal endothelin cascade in the hyperpigmentation mechanism of lentigo senilis. J. Investig. Dermatol.

[13] Hattori H., Kawashima M., Ichikawa Y., Imokawa G. (2004). The epidermal stem cell factor is over-expressed in lentigo senilis: Implication for the mechanism of hyperpigmentation. J. Investig. Dermatol.

[14] Kang H.Y., Hwang J.S., Lee J.Y., Ahn J.H., Kim J.Y., Lee E.S., Kang W.H. (2006). The dermal stem cell factor and c-kit are overexpressed in melasma. Br. J. Dermatol.

[15] Imokawa G., Yada Y., Morisaki N., Kimura M. (1998). Biological characterization of human fibroblast-derived mitogenic factors for human melanocytes. Biochem. J.

[16] Chakraborty A.K., Funasaka Y., Slominski A., Ermak G., Hwang J., Pawelek J., Ichihashi M. (1996). Production and release of proopiomelanocortin (POMC) derived peptides by human melanocytes and keratinocytes in culture: Regulation by ultraviolet B. Biochim. Biophys. Acta.

[17] Schauer E., Trautinger F., Köck A., Schwarz A., Bhardwaj R., Simon M., Ansel J.C., Schwarz T., Luger T.A. (1994). Proopiomelanocortin-derived peptides are synthesized and released by human keratinocytes. J. Clin. Investig.

[18] Abdel-Malek Z., Swope V.B., Suzuki I., Akcali C., Harriger M.D., Boyce S.T., Urabe K., Hearing V.J. (1995). Mitogenic and melanogenic stimulation of normal human melanocytes by melanotropic peptides. Proc. Natl. Acad. Sci. USA.

[19] Suzuki I., Cone R.D., Im S., Nordlund J., Abdel-Malek Z.A. (1996). Binding of melanotropic hormones to the melanocortin receptor MC1R on human melanocytes stimulates proliferation and melanogenesis. Endocrinology.

[20] Kawashima M., Imokawa G. (2005). Hyperpigmentation mechanisms involved in UVB-melanosis and solar lentigo and clinical effects of Chamomira ET extract on the pigmentation. Mon. Book Derma.

[21] Kobayashi T., Imokawa G., Hearing V.J. (1996). Differentiation of epidermal melanocytes by UVB irradiation in tail skin of mouse (C57BL/6J mice-aa/ee, recessive yellow) with mutation of melanocortin receptor. Jpn. J. Dermatol.

[22] Niwano T., Nakajima H., Imokawa G. (2012). Paracrine interaction between UVB-exposed human keratinocytes and human melanocytes in co-culture system with cell insert leading to an increased expression of tyrosinase and its blockade by *Witherferin* A. Pigment Cell Melanoma Res.

[23] Kolch W., Heidecker G., Kochs G., Hummel R., Vahidi H., Mischak H., Finkenzeller G., Marme D., Rapp U.R. (1993). Protein kinase Cα activates RAF-1 by direct phosphorylation. Nature.

[24] Schonwasser D.C., Marais R.M., Marshall C.J., Parker P.J. (1998). Activation of the mitogen-activated protein kinase/extracellular signal-regulated kinase pathway by conventional, novel, and atypical protein kinase C isotypes. Mol. Cell. Biol.

[25] Mason C.S., Springer C.J., Cooper R.G., Superti-Furga G., Marshall C.J., Marais R. (1999). Serine and tyrosine phosphorylations cooperate in Raf-1, but not B-Raf activation. EMBO J.

[26] Marais R., Light Y., Mason C., Paterson H., Olson M.F., Marshall C.J. (1998). Requirement of Ras-GTP-Raf complexes for activation of Raf-1 by protein kinase C. Science.

[27] Imokawa G., Kobayashi T., Miyagishi M. (2000). Intracellular signaling mechanisms leading to synergistic effects of endothelin-1 and stem cell factor on proliferation of cultured human melanocytes: Cross-talk via trans-activation of the tyrosine kinase c-kit receptor. J. Biol. Chem.

[28] Sato-Jin K., Nishimura E.K., Akasaka E., Huber W., Nakano H., Miller A., Du J., Wu M., Hanada K., Sawamura D. (2008). Epistatic connections between MITF and endothelin signaling in Waardenburg syndrome and other pigmentary disorders. FASEB J.

[29] Blume-Jensen P., Claesson-Welsh L., Siegbahn A., Zsebo K.M., Westermark B., Heldin C.H. (1991). Activation of the human c-kit product by ligand-induced dimerization mediates circular actin reorganization and chemotaxis. EMBO J.

[30] Cutler R.L., Liu L., Damen J.E., Krystal G. (1993). Multiple cytokines induce the tyrosine phosphorylation of Shc and its association with Grb2 in hemopoietic cells. J. Biol. Chem.

[31] Lennartsson J., Blume-Jensen P., Hermanson M., Ponten E., Carlberg M., Ronnstrand L. (1999). Phosphorylation of Shc by Src family kinases is necessary for stem cell factor receptor/c-kit mediated activation of the Ras/MAP kinase pathway and c-fos induction. Oncogene.

[32] Liu L., Damen J.E., Cutler R.L., Krystal G. (1994). Multiple cytokines stimulate the binding of a common 145-kilodalton protein to Shc at the Grb2 recognition site of Shc. Mol. Cell. Biol.

[33] Wan P., Hu Y., He L. (2011). Regulation of melanocyte pivotal transcription factor MITF by some other transcription factors. Mol. Cell. Biochem.

[34] Potterf S.B., Furumura M., Dunn K.J., Arnheiter H., Pavan W.J. (2000). Transcription factor hierarchy in Waardenburg syndrome: Regulation of MITF expression by SOX10 and PAX3. Hum. Genet.

[35] Bentley N.J., Eisen T., Goding C.R. (1994). Melanocyte-specific expression of the human tyrosinase promoter: Activation by the microphthalmia gene product and role of the initiator. Mol. Cell. Biol.

[36] Fang D., Setaluri V. (1999). Role of microphthalmia transcription factor in regulation of melanocyte differentiation marker TRP-1. Biochem. Biophys. Res. Commun.

[37] Du J., Miller A.J., Widlund H.R., Horstmann M.A., Ramaswamy S., Fisher D.E. (2003). MLANA/MART1 and SILV/PMEL17/GP100 are transcriptionally regulated by MITF in melanocytes and melanoma. Am. J. Pathol.

[38] Mizutani Y., Hayashi N., Kawashima M., Imokawa G. (2010). A single UVB exposure increases the expression of functional KIT in human melanocytes by up-regulating MITF expression through the phosphorylation of p38/CREB. Arch. Dermatol. Res.

[39] Nakajima H., Wakabayashi Y., Wakamats K., Imokawa G. (2011). An extract of *Melia toosendan* attenuates endothelin-1-stimulated pigmentation in human epidermal equivalents through the interruption of PKC activity within melanocytes. Arch. Dermatol. Res.

[40] Nakajima H., Fukazawa K., Wakabayashi Y., Wakamatsu K., Imokawa G. (2012). WSE attenuates stem cell factor-stimulated pigmentation in human epidermal equivalent through interruption of ERK phosphorylation within melanocytes. J. Nat. Med.

[41] Nakajima H., Wakabayashi Y., Wakamatsu K., Imokawa G. (2011). An extract of *Withania somnifera* attenuates endothelin-1-stimulated pigmentation in human epidermal equivalents through the interruption of PKC activity within melanocytes. Phytother. Res.

[42] Nakajima H., Fukazawa K., Wakabayashi Y., Wakamatsu K., Senda K., Imokawa G. (2012). Abrogating effect of a xanthophyll carotenoid astaxanthin on the stem cell factor-induced stimulation of human epidermal pigmentation. Arch. Dermatol. Res.

[43] Agarwal R., Diwanay S., Patki P., Patwardhan B. (1999). Studies on immunomodulatory activity of *Withania somnifera* (Ashwagandha) extract in experimental immune inflammation. J. Ethnopharmacol.

[44] Gupta S.K., Mohanty I., Talwar K.K., Dinda A., Joshi S., Bansal P., Saxena A., Arya D.S. (2004). Cardioprotection from ischemia and reperfusion injury by *Withania somnifera*: A hemodynamic, biochemical and histopathological assessment. Mol. Cell. Biochem.

[45] Ahmad M., Saleem S., Ahmad A.S., Ansari M.A., Yousuf S., Hoda M.N., Islam F. (2005). Neuroprotective effects of *Withania somnifera* on 6-hydroxydopamine induced Parkisonism in rats. Hum. Exp. Toxicol.

[46] Owais M., Sharad K.S., Shehbaz A., Saleemuddin M. (2005). Antibacterial efficacy of *Withania somnifera* (Ashwagandha) an indigenous medicinal plant against experimental murine salmonellosis. Phytomedicine.

[47] Rasool M., Varalakshmi P. (2006). Immunomodulatory role of *Withania somnifera* root powder on experimental induced inflammation: An *in vivo* and *in vitro* study. Vascul. Pharmacol.

[48] Singh D., Aggarwal A., Maurya R., Naik S. (2007). *Withania somnifera* inhibits NF-κB and AP-1 transcription factors in human peripheral blood and synovial fluid mononuclear cells. Phytother. Res.

[49] Ichikawa H., Takad Y., Shishodia S., Jayaprakasam B., Nair M.G., Aggarwal B.B. (2006). Withanolides potentiate apoptosis, inhibit invasion, and abolish osteoclastogenesis through suppression of nuclear factor-κB (NF-κB) activation and NF-κB-regulated gene expression. Mol. Cancer Ther.

[50] Kaileh M., van den Berghe W., Heyerick A., Horion J., Piette J., Libert C., de Keukeleire D., Essawi T., Haegeman G. (2007). *Withaferin* A strongly elicits IκB kinase β hyperphosphorylation concomitant with potent inhibition of its kinase activity. J. Biol. Chem.

[51] Aalinkeel R., Hu Z., Nair B.B., Sykes D.E., Reynolds J.L., Mahajan S.D., Schwartz S.A. (2010). Genomic analysis highlights the role of the JAK-STAT signaling in the anti-proliferative. Altern. Med.

[52] Terazawa S., Nakajima H., Niwano T., Wakabayashi Y., Fukazawa K., Imokawa G. (2013). *Withaferin* A attenuates SCF-stimulated pigmentation in human epidermal equivalents by interrupting c-KIT activation within human melanocytes. Pigment Cell Melanoma Res.

